# Knowledge, attitudes and practices towards people living with HIV/AIDS in Lebanon

**DOI:** 10.1371/journal.pone.0249025

**Published:** 2021-03-25

**Authors:** Lara Youssef, Souheil Hallit, Hala Sacre, Pascale Salameh, Michelle Cherfan, Marwan Akel, Mira Hleyhel

**Affiliations:** 1 Department of Medicine and Medical sciences, University of Balamand (UOB), Al-Kurah, Lebanon; 2 Faculty of Medicine and Medical Sciences, Holy Spirit University of Kaslik (USEK), Jounieh, Lebanon; 3 INSPECT-LB: National Institute of Public Health, Clinical Epidemiology and Toxicology, Beirut, Lebanon; 4 Faculty of Pharmacy, Lebanese University, Beirut, Lebanon; 5 Faculty of Medicine, University of Nicosia, Nicosia, Cyprus; 6 School of Pharmacy, Lebanese International University, Beirut, Lebanon; 7 Faculty of Public Health, Lebanese University, Fanar, Lebanon; 8 Faculty of Public Health, CERIPH, Center for Research in Public Health—Pharmacoepidemiology Surveillance Unit, Lebanese University, Fanar, Lebanon; Hofstra University, UNITED STATES

## Abstract

**Background:**

Up till today, studies carried in Lebanon have focused more on the prevalence of HIV and behaviors and quality of life of infected individuals, however, none of these studies discussed the degree of stigma towards these populations. Therefore, the aim of this study is to measure the rate of stigma in terms of knowledge, attitude and practice towards patients living with Human Immunodeficiency Virus (HIV) and Acquired Immunodeficiency Syndrome (AIDS) (PLWHA) and examine the factors associated with this stigma.

**Methods:**

A cross-sectional survey, enrolling 862 participants, was carried across the five governorates in Lebanon: Beirut, Mount Lebanon, North, South and Bekaa. The survey was a self-administered questionnaire which covered information about participants’ general demographics, their knowledge, attitudes, practices and awareness towards HIV/AIDS in Lebanon., attitudes towards PLWHA, practices related to HIV/AIDS and awareness regarding HIV/AIDS situation in Lebanon.

**Results:**

The response rate to the survey was 78.36% (862 participants). Being Muslim (Beta = -2.56) or Druze (Beta = -2.64) compared to Christians were significantly associated with lower knowledge towards HIV/AIDS, whereas having a secondary (Beta = 2.71) and a university (Beta = 3.04) levels of education compared to illiteracy and higher age (Beta = 0.05) were significantly associated with higher knowledge. Higher knowledge (Beta = 0.66) was significantly associated with better attitude, whereas higher age (Beta = -0.14) and being Muslim compared to Christian (Beta = -3.44) were significantly associated with worse attitude. Better attitude (Beta = 0.02) was significantly associated with better practice, whereas females compared to males (Beta = -0.39), having a secondary level of education compared to illiteracy (Beta = -0.88) and being Muslim compared to Christian (Beta = -0.32) were significantly associated with worse practice.

**Conclusion:**

Our results stress the need for educational programs, advocacy campaigns and policies to help reduce HIV stigma. This will then help start developing interventions and strategies for a possible reduction in the stigmatization level.

## Background

Human Immunodeficiency Virus (HIV) infection and Acquired Immune Deficiency Syndrome (AIDS) remain a major public health problem [1]. According to the latest statistics on the status of the AIDS epidemic, it was reported that 36.9 million people globally were living with HIV in 2017 [2]. While the number of HIV cases in the Middle East and North Africa (MENA) region is low compared to other regions, recent studies showed that HIV incidence is increasing especially among high-risk populations [3]. In Lebanon, the most recent published data from UNAIDS reported the number of HIV cases in 2017 to be 2200 cases [4]. This number is thought to be higher in reality due to under-detection and under-reporting of incident cases of HIV in Lebanon [5,6]. It is important to highlight here that the majority of HIV cases in Lebanon are primarily through Men who have sex with men (MSM) and less frequently through other risk populations like injecting drug users (IDU) and prisoners [7].

One of the corner stone in fighting HIV/AIDS is understanding the knowledge, attitudes and practices (KAP) of people towards it. The poor level of knowledge or the miseducation about HIV/AIDS of the general population is the most common problem people living with HIV/AIDS (PLWHA) are facing; it mostly results in discrimination against them [8] that leads to or precipitates problems with disclosure, social isolation, access to antiretroviral therapy and psychological support [9–11]. It also negatively impacts the health, quality of life, social support and well-being of people living with PLWHA. Although very few studies have touched upon this in Lebanon and in the region, we assume that similar problems manifest themselves here.

Moreover, several factors are associated with HIV knowledge level and play an important role in determining the level of HIV stigma. These factors are mainly education and attitudes towards HIV. Increasing education and awareness levels about HIV are two strategies, among many more other effective strategies, to reducing the degree of stigma. Consequently, when education level increases, knowledge about HIV increases, negative attitudes towards PLWHA decrease, and thus HIV stigma level decreases [12,13]. This is supported by several quasi-experimental studies that explored the effect of educational and awareness interventions on HIV stigma [14,15]. For example, a study in Canada showed that increasing participants’ awareness about HIV helped shape them into influence champions in their communities, and consequently reduced HIV stigma [14]. Moreover, another study in a more sensitive culture; in Egypt, authors’ educational and awareness intervention in a healthcare setting proved to reduce HIV stigma and discrimination [15]. Finally, most of the US Centers for Disease Control and Prevention’s (CDC) activities are focused on interventions that entangle public education activities and social marketing campaigns [16].

Additional factor associated with HIV stigma was the economical level, where employed community members had more supportive attitudes towards HIV/AIDS [17]. It is believed that giving the right amount of knowledge helps foster positive attitudes towards PLWHA and consequently motivates the population towards adapting positive and safe practices. Thus, improving the level of knowledge is very important and might help control and reduce HIV incidence. The more people are educated about the virus and its route of transmission, the less they would engage in risky behaviors and the less judgmental they act towards PLWHA [18]. Moreover, the younger generation represents the next generation on which HIV prevention should be most focused on; thus investment should target this generation to promote safe sexual practices [19].

Despite the decrease in mortality and improvement in quality of life that resulted from the introduction of the highly active antiretroviral therapy, HIV remains a controversial topic and a taboo in the MENA region [20]. Data from the region still show low level of knowledge that is associated with higher level of stigma [21]. For example, a study among university students in the United Arab Emirates revealed alarming gaps in knowledge and high levels of fear and intolerance towards PLWHA [22]. In Lebanon, on the other hand, only few studies were conducted on KAP and showed that there was low awareness and low knowledge of protection. This indicates that misconceptions are still prevailing in the community, which may be the factor leading to negative attitudes towards PLWHA [23].

Based on the aforementioned, it is very important to clearly understand the level of KAP toward HIV/AIDS and how people’s sociodemographic background affects it, to better plan for adequate and appropriate awareness and prevention programs [24]. Therefore, the aim of this study was to evaluate the level of knowledge, attitudes and practices towards HIV/AIDS among the general population in Lebanon.

## Methods

### Ethics approval and consent to participate

Ethical approval was obtained from the Lebanese National AIDS Committee and the Research Committee on Ethics at the Lebanese International University (LIU). All participants provided a written informed consent after receiving the study information. Data confidentiality and anonymity were assured.

### Study design and participants

This cross-sectional study was conducted between September 2018 and February 2019 using convenience sampling in the five Lebanese governorates (Mount Lebanon, Beirut, North, South and Bekaa) to recruit individuals aged 18 years and above. Participants were actively approached in different public spaces, such as roads, shopping malls, restaurants, universities, etc. to fill out a self-administered questionnaire. For participants’ who were illiterate, they were offered assistance in filling out the survey, to ensure that everyone gets an equal opportunity to participate in the study.

### Minimal sample size calculation

To determine the study sample size, we reviewed recent publication in Lebanon exploring HIV knowledge. It was observed that the level of knowledge has been regressing to reach 85.7% in 2004 [23]. Taking into consideration the conclusion of the latter report, and since there is no recent study that has evaluated the knowledge of HIV among the general population, we used 50% which is conservative and gives the largest sample size. Based on this prevalence, an alpha risk of 5% and a margin of error of 5%, the target sample size was 384 individuals. The sample size calculation was performed on Epi Info.

### Data collection

A questionnaire was developed in English with questions adapted from similar previous studies that used validated scales to measure people’s knowledge about HIV, attitudes towards PLWHA and the level of stigma [25–27]. The questionnaire was later translated into Arabic and then translated back into English to check for compatibility. The survey was reviewed many times by the research team then piloted in a sample of 25 participants. It is important to mention here that questions were adapted in a way that took into consideration the Lebanese culture and social norms.

Internal consistency of items was checked using Cronbach’s alpha, where similar questions from different categories were compared for reliability. An exploratory factor analysis was conducted to confirm the construct measured by each category. The results of these analyses can be viewed in S1 to S4 Tables.

The survey collected information on general demographics including age, sex, governorate, occupation, income, etc., HIV/AIDS general knowledge, HIV/AIDS mode of transmission knowledge, protection knowledge, attitudes towards PLWHA, practices related to HIV/AIDS and awareness regarding HIV/AIDS situation in Lebanon.

### Scoring

A knowledge score, an attitude score and a practice score were calculated for each participant.

#### HIV/AIDS knowledge score

This section included 62 questions about Knowledge which were divided into sub-categories: general knowledge, transmission knowledge and protection knowledge, each category constituted of 20, 23 and 19 questions, respectively. Answer options were coded as 0 = “No”, 1 = “Yes” and 2 = “Don’t know”. For scoring calculation, a correct answer was computed as 1 and a wrong answer was computed as 0. Not answering or not knowing was considered as neutral and calculated as 0. For the general knowledge questions, the proportion of participants who responded as “Don’t know” was 0.22 (22%). The range was between 1.6% and 49%, which corresponded to the following questions: “Is AIDS a sexually transmitted disease?” and “Can HIV infection develop into AIDS within a year?”, respectively. For the transmission questions, the proportion of participants who answered “Don’t Know” was 0.098 (9.8%). The range was between 1.1% and 29% and these corresponded to the following questions: “Unprotected sexual intercourse cases transmission” and “Sexual intercourse while the female is taking contraceptives causes transmission”, respectively. Finally, the proportion of participants who responded as “Don’t Know” for the protection questions was 0.072 (7.2%). The range was between 1.4% and 21.5% for the following questions: “Taking a blood test before marriage is protective” and “Doing a sexual intercourse while the female is taking contraceptive is protective”, respectively.

Scores for each knowledge group was computed by summing scores of all items. Second, these scales were categorized into two groups: low/medium level category and high-level category. The cut off points used to categorize each category is found in *S1 Appendix*. For general knowledge, a score between 0 and 13 was considered low/medium category and a score of 14 to 20 was considered high category. For transmission knowledge, a score between 0 and 15 was considered low/medium category and a score of 16 to 23 was considered high category. For protection knowledge, a score between 0 and 13 was considered low/medium category and a score of 14 to 19 was considered high category. For total knowledge, which was the sum of general, transmission and protection knowledge, a score between 0 and 41 was considered as low/medium category and a score of 42 to 62 was considered as high category. Please refer to supplementary figures for the distribution of scales for each category.

#### Attitudes towards PLWHA score

This section included 42 statements that investigated participants’ attitudes and discrimination towards PLWHA and the degree of stigmatization. The answer options were on a 4-point Likert-scale that ranged from strongly disagree to strongly agree. An answer of strongly disagree or disagree was calculated as 0, and agree or strongly agree as 1. A higher mean score indicates more positive attitudes.

#### Practices related to HIV/AIDS score

This section included 14 questions which aimed at better understanding participants’ practices, specifically sexual practices related to HIV. Answer options to these questions were coded as 0 = “No”, 1 = “Yes”, 2 = “Don’t know” and 3 = “Not applicable”. For scoring calculation, a correct answer was computed as 1, wrong answer as 0. Not answering or not knowing was considered as neutral and calculated as 0. The proportion of participants who responded as “Don’t Know” to practices questions was 0.035 (3.5%) and the range was between 0.7% and 10.8%, and these were for the questions: “I share needles with other drug users” and “I know my HIV status”, respectively. As for those who responded as “Not Applicable”, the proportion was 0.3 (30%) and the range was between 1.4% and 53.7% for the questions: “I have had sexual intercourse before” and “I use a condom regularly during sexual intercourse with occasional partner”, respectively.

### Statistical analysis

After collection, data were entered into an Excel database then analyzed using SPSS version 24. A polychoric correlation was run using the principal component analysis technique for the knowledge, attitude and practice items using the FACTOR software. Since the extracted factors were not found to be highly correlated, the varimax rotation was used. The Kaiser-Meyer-Olkin (KMO) measure of sampling adequacy and Bartlett’s test of sphericity were calculated to ensure sample’s adequacy. Factors with an Eigen value >1 were retained. When the sample size is sufficiently large (>200), the normality assumption is not needed at all as the Central Limit Theorem ensures that the distribution of disturbance term will approximate normality [28]. The independent-sample t-test was used when comparing two means, whereas the ANOVA test was used to compare 3 means or more. The Pearson’s correlation coefficient was used to test linear association between 2 quantitative/continuous variables. Since better knowledge would lead to better attitudes, which would lead to better practice, three stepwise linear regressions were conducted; in the first one, the knowledge score was the dependent variable and the sociodemographic variables the independent ones. In the second one, the attitude score was the dependent variable, with the sociodemographic variables and the knowledge score the independent ones. Finally, in the third regression, the practice score was the dependent variable and the sociodemographic variables, knowledge and attitude scores the independent variables. All variables that showed a p<0.2 in the bivariate analysis were taken as independent variables in the regression model in order to eliminate as much as possible the potential confounding factors. Moreover, Cronbach’s alpha was recorded for reliability analysis for all the scales. All analyses were carried at 0.05 level of significance.

## Results

### Reliability of the scales

The reliability analysis results showed Cronbach’s alpha values for the three scales as follows: knowledge (0.813), attitude (0.887) and practice (0.676).

### Response rate

Out of the 1100 approached participants, 862 responded to the survey. The response rate was 78.36%.

### Sociodemographic characteristics of the participants

The sociodemographic and other characteristics of the participants are summarized in Table 1. The mean age of the participants was 31.31 ± 13.33 years, with 55.4% females. The majority were single (62.7%), with a university level of education (72.7%) and living in the Mount Lebanon (49.3%).

**Table 1:** Sociodemographic and other characteristics of the participants (N = 862).

Variable	Mean ± SD/N (%)
Age (in years)	31.31 ± 13.33
Gender	
**Male**	382 (44.6%)
**Female**	474 (55.4%)
Marital status	
**Married**	282 (33.1%)
**Single**	535 (62.7%)
**Divorced**	15 (1.8%)
**Widowed**	13 (1.5%)
Education	
**Illiteracy**	17 (2.0%)
**Primary**	13 (1.5%)
**Complementary**	38 (4.5%)
**Secondary**	100 (11.9%)
**Technical**	62 (7.4%)
**University**	613 (72.7%)
Religion	
**Christian**	388 (46.7%)
**Muslim**	364 (43.9%)
**Druze**	66 (8.0%)
**Other**	12 (1.4%)
District	
**Beirut**	321 (41.0%)
**Mount Lebanon**	386 (49.3%)
**North**	28 (3.6%)
**South**	37 (4.7%)
**Bekaa**	11 (1.4%)
Monthly income	
**No income**	21 (2.6%)
**<1000 USD**	174 (21.2%)
**1000–1500 USD**	244 (29.7%)
**1501–3000 USD**	239 (29.1%)
**>3000 USD**	143 (17.4%)

Abbreviations: SD = Standard Deviation, USD = United States Dollar.

### Description of knowledge scales

A general distribution of participants’ answers for each knowledge category were computed based on the cut off points stated earlier. For general knowledge, the majority of participants had a low/medium level of knowledge (67.8%), while for transmission and protection knowledge the majority had high level of knowledge (75.7% and 71.6% respectively). When total knowledge was computed, the majority of participants belonged to the high-level knowledge (68.1%). Regarding attitudes and practices, the majority of participants belonged to the low/medium category group (59.8% and 52% respectively). Similarly, regarding awareness about the HIV/AIDS situation in Lebanon, 94.6% of participants belonged to the low/medium category group. These results can be viewed in *Table 2*.

**Table 2:** Distribution of participants’ answer in each category based on cut-off point.

Category	Low/Medium N (%)	High N (%)
**General knowledge**	287 (67.8)	136 (32.2)
**Transmission knowledge**	103 (24.3)	320 (75.7)
**Protection knowledge**	120 (28.4)	303 (71.6)
**Total knowledge**	135 (31.9)	288 (68.1)

### Description of the attitude and practice scores

The mean knowledge score was 38.49 ± 7.15, mean attitude score 114.55 ± 15.78 and that of the practice score 2.2 ± 1.59. In the absence of cutoff values for each scale, the median was considered as the cutoff point; the results showed that 462 (53.6%) had adequate knowledge, 458 (53.1%) had adequate attitudes, and 443 (51.4%) had good practices towards patients with HIV/AIDS.

### Factor analysis

The results of the factor analysis are summarized in S1–S4 Tables. None of the items was removed from the knowledge or attitude scales. The KMO and Bartlett’s p values showed sample adequacy for both scales.

### Factors associated with knowledge, attitudes and practices

The bivariate analysis of factors associated with the knowledge, attitude and practice of the participants regarding HIV are summarized in Table 3. Concerning knowledge, a significantly higher knowledge score was seen in participants with a university level of education compared to all other categories, in Christians compared to other religions, in those living in Mount Lebanon compared to other districts and in those whose monthly income is >3000 USD compared to all other categories. Furthermore, a significantly higher mean knowledge score was seen in married compared to single, divorced or widowed participants.

**Table 3:** Bivariate analysis of factors associated with the knowledge, attitude and practice of the participants regarding HIV.

Variable	Knowledge	Attitude	Practice
Gender			
**Male**	38.12 ± 7.28	114.00 ± 15.73	2.60 ± 1.53
**Female**	38.80 ± 7.00	115.01 ± 15.89	2.26 ± 1.62
**p-value**	0.166	0.357	**0.002**
Education level			
**Illiterate**	32.11 ± 7.08	119.47 ± 17.43	3.14 ± 1.87
**Primary**	38.00 ± 6.96	120.53 ± 12.58	2.20 ± 1.31
**Complementary**	37.10 ± 7.56	109.60 ± 12.93	1.55 ± 1.34
**Secondary**	38.68 ± 6.42	112.29 ± 16.36	2.70 ± 1.58
**Technical**	37.41 ± 7.83	112.43 ± 14.41	2.22 ± 1.61
**University**	38.88 ± 7.11	115.27 ± 16.01	2.43 ± 1.58
**p-value**	**0.003**	**0.041**	**0.003**
Religion			
**Christian**	39.74 ± 5.92	116.41 ± 15.23	2.51 ± 1.53
**Muslim**	37.42 ± 7.83	112.38 ± 15.83	2.23 ± 1.62
**Druze**	37.57 ± 7.58	113.72 ± 14.34	2.56 ± 1.61
**Others**	38.83 ± 7.61	124.16 ± 20.22	3.18 ± 1.47
**p-value**	**<0.001**	**0.001**	**0.026**
District			
**Beirut**	38.02 ± 7.11	113.82 ± 16.90	2.44 ± 1.56
**Mount Lebanon**	39.47 ± 6.60	115.97 ± 15.11	2.50 ± 1.56
**North**	36.92 ± 6.98	113.21 ± 13.65	2.55 ± 1.55
**South**	39.10 ± 4.99	111.78 ± 16.00	1.89 ± 1.74
**Bekaa**	35.54 ± 7.11	111.18 ± 16.55	3.20 ± 1.39
**p-value**	**0.014**	0.242	0.114
Monthly income			
**No income**	38.90 ± 5.99	117.76 ± 8.84	1.05 ± 1.50
**<1000 USD**	36.63 ± 9.23	113.43 ± 15.68	2.47 ± 1.68
**1000–1500 USD**	38.44 ± 6.52	114.61 ± 15.64	2.44 ± 1.54
**1501–3000 USD**	39.02 ± 6.30	114.38 ± 15.05	2.35 ± 1.58
**>3000 USD**	40.00 ± 5.87	116.11 ± 18.99	2.77 ± 1.44
**p-value**	**<0.001**	0.547	**<0.001**
Marital status			
**Married**	39.91 ± 5.45	113.31 ± 14.33	2.31 ± 1.48
**Single**	37.69 ± 7.85	115.36 ± 16.28	2.45 ± 1.65
**Divorced**	39.46 ± 5.93	110.66 ± 9.22	1.93 ± 2.01
**Widowed**	39.15 ± 4.33	112.38 ± 22.78	2.80 ± 1.40
**p-value**	**<0.001**	0.228	0.349

• Posthoc analysis knowledge score: Marital status (married vs single p<0.001); Education level (illiteracy vs secondary p = 0.007; illiteracy vs university p = 0.002); religion (christian vs muslim p<0.001); Mouhafaza (Beirut vs Mount Lebanon p = 0.049); monthly income (<1000 USD vs 1501–3000 USD p = 0.007; <1000 USD vs >3000 USD p<0.001).

• Posthoc analysis attitude score: Religion (christian vs muslim p = 0.002).

• Post hoc analysis practice score: Education level (illiteracy vs complementary p = 0.023; complementary vs university p = 0.02; complementary vs secondary p = 0.003); monthly income (no income vs <1000 USD p = 0.001; no income vs 1000–1500 USD p = 0.002; no income vs 1501–3000 USD p = 0.004; no income vs >3000 USD p<0.001).

Regarding attitude, a significantly higher mean attitude score was seen in those with a primary level of education compared to other categories and in those from a religion other than Christians, Muslims or Druze. As for practice, a significantly higher practice score was seen in males compared to females (2.60 vs 2.26), in illiterate participants compared to other education levels, in those from a religion other than Christians, Muslims or Druze and in those whose monthly income is >3000 USD compared to all other categories. Higher knowledge was significantly but weakly associated with higher attitude (r = 0.3), higher practice (r = 0.097) and older age (r = 0.101). Finally, higher attitude was significantly but weakly associated with higher practice (r = 0.147) (Table 4).

**Table 4:** Correlation of continuous variables with the knowledge, attitude and practice of the participants regarding HIV.

Variable	Knowledge	Attitude	Practice
Knowledge			
**r**	1		
**p-value**	-		
Attitude			
**r**	0.3	1	
**p-value**	**<0.001**	-	
Practice			
**r**	0.097	0.147	1
**p-value**	**0.005**	**<0.001**	-
Age			
**r**	0.101	-0.067	-0.061
**p-value**	**0.005**	0.064	0.095

### Regression analyses of factors associated with knowledge, attitudes and practices

A first linear regression, taking the knowledge score as the dependent variable, showed that being Muslim (Beta = -2.56) or Druze (Beta = -2.64) compared to Christians were significantly associated with lower knowledge towards HIV/AIDS, whereas having a secondary (Beta = 2.71) and a university (Beta = 3.04) levels of education compared to illiteracy and higher age (Beta = 0.05) were significantly associated with higher knowledge scores (Table 5, Model 1).

**Table 5:** Multivariable analysis.

Model 1: Linear regression taking the knowledge score as the dependent variable.				
Variable	**Unstandardized Beta**	**Standardized Beta**	**p-value**	**95% Confidence Interval**
**Muslim compared to Christian**	-2.56	-0.183	<0.001	-3.623 - -1.494
**Druze compared to Christian**	-2.64	-0.103	0.008	-4.564 - -0.705
**University level of education compared to illiteracy**	3.04	0.199	<0.001	1.600–4.488
**Secondary level of education compared to illiteracy**	2.71	0.128	0.005	0.807–4.613
**Age**	0.05	0.097	0.014	0.010–0.093
Model 2: Linear regression taking the attitude score as the dependent variable.				
Variable	**Unstandardized Beta**	**Standardized Beta**	**p-value**	**95% Confidence Interval**
**Knowledge score**	0.66	0.292	<0.001	0.503–0.818
**Age**	-0.14	-0.116	0.001	-0.225 - -0.056
**Muslim compared to Christian**	-3.44	-0.107	0.003	-5.692 - -1.192
Model 3: Linear regression taking the practice score as the dependent variable.				
Variable	**Unstandardized Beta**	**Standardized Beta**	**p-value**	**95% Confidence Interval**
**Attitude score**	0.02	0.138	<0.001	0.007–0.022
Gender (females vs males*)	-0.39	-0.125	0.001	-0.626–0.158
**Secondary level of education compared to illiteracy**	-0.88	-0.119	0.002	-1.423 - -0.330
**Muslim compared to Christian**	-0.32	-0.102	0.008	-0.558 - -0.086

• Variables entered in model 1: Age, gender, education level, religion, income.

• Variables entered in model 2: Age, gender, education level, religion, knowledge score.

• Variables entered in model 3: Age, gender, education level, religion, income, knowledge score, attitude score.

A second linear regression, taking the attitude score as the dependent variable, showed that higher knowledge (Beta = 0.66) was significantly associated with better attitude, whereas higher age (Beta = -0.14) and being Muslim compared to Christian (Beta = -3.44) were significantly associated with worse attitude (Table 5, Model 2).

A third linear regression, taking the practice score as the dependent variable, showed that a better attitude (Beta = 0.02) was significantly associated with better practice, whereas females compared to males (Beta = -0.39), having a secondary level of education compared to illiteracy (Beta = -0.88) and being Muslim compared to Christian (Beta = -0.32) were significantly associated with worse practice (Table 5, Model 3).

### Awareness about HIV/AIDS

The descriptive analysis concerning awareness about HIV/AIDS showed that 265 (31%) and 462 (54.0%) of the participants discussed HIVS/AIDS related topics with their parents and friends respectively. In addition, 123 (14.4%) have ever checked the website of the Ministry of Public Health of Lebanon for HIV/AIDS information, and 87 (10.2%) know an association for HIV/AIDS in Lebanon (Table 6).

**Table 6:** Description of awareness level.

Variable	N (%)
**Ever discussed HIV/AIDS related topics with your parents (yes)**	265 (31.0%)
**Ever discussed HIV/AIDS related topics with your friends (yes)**	462 (54.0%)
**Know anyone with HIV/AIDS (yes)**	121 (14.2%)
**Know someone who died from HIV/AIDS (yes)**	133 (15.6%)
**Discuss the risks with your partner (yes)**	350 (41.3%)
**Think HIV/AIDS is still a taboo in Lebanon (yes)**	552 (65.2%)
**Seek counseling and advice if you suspected having HIV/AIDS (yes)**	742 (87.2%)
**My professional education has provided me with enough education/information to work safely with PLWHA (yes)**	262 (30.9%)
**Using condoms is necessary during sexual intercourse with occasional partner (yes)**	746 (88.7%)
**Everyone should ask their partner to test for HIV before the first sexual intercourse (yes)**	721 (84.8%)
**Belief that HIV/AIDS campaigns are adequately/frequently conducted in Lebanon (yes)**	298 (34.9%)
**More awareness campaigns on HIV/AIDS are needed among the Lebanese population (yes)**	766 (89.6%)
**We should advocate for rights of PLWHA (yes)**	740 (86.8%)
**We should offer emotional, physical and referral support for PLWHA (yes)**	721 (84.6%)
**Ever checked the website of the Ministry of Public Health of Lebanon for HIV/AIDS information (yes)**	123 (14.4%)
**Know any association for HIV/AIDS in Lebanon**	87 (10.2%)

Abbreviations: AIDS, HIV, PLWHA.

## Discussion

A total of 856 participants answered the present survey that gives a deeper insight into the social factors associated with knowledge and attitudes inclination. Specifically, multivariable models revealed that being Christian or having a higher level of education were associated with higher level of knowledge. Being Christian, younger, and having higher HIV knowledge were associated with positive attitudes towards PLWHA. Also, negative practices were associated with having negative attitudes towards PLWHA, being a Muslim, being a female and having a secondary level education.

### HIV knowledge

Comparing our results to similar studies done in the MENA region, the Lebanese population is privileged with a higher level of knowledge. The majority of our participants were able to identify correct answers to statements regarding HIV. While in a study done with a similar population in the Kingdom of Saudi Arabia (KSA), the majority of participants showed an unsatisfactory level of knowledge [21]. Furthermore, studies in Iran and KSA have significantly shown that the level of participants’ knowledge was associated with their attitudes towards HIV [21,29]. This is in agreement with our results, where an increased level of knowledge was associated with more positive attitudes. One major difference between Lebanon and these countries is the cultural and religious beliefs. Lebanon is known to have more freedom when it comes to cultural and religious beliefs, in addition to increased adoption of voluntary HIV testing among vulnerable population, which explains why participants had more positive attitudes compared with participants from the KSA and Iran where rules are more severe [7].

### Attitudes towards PLWHA

Moving on to factors that were associated with knowledge level and attitudes, these played an important role in determining the level of stigmatization. People who are less educated and from a family with lower income have less exposure to diverse groups of people. On another hand, people with higher education and income are able to comprehend and foster people with HIV since they are more aware of modes of transmission and management strategies [30]. Although these results are completely compatible with those in the literature, they do come in line with studies reporting on income, where economic level and employability stand as two strong elements in determining knowledge level and thus supporting positive attitudes towards HIV/AIDS [12,13,17,31].

Being less knowledgeable about the routes of HIV transmission, affects people’s attitudes towards PLWHA. Thus, negative attitudes are triggered when people are driven by the misjudgment of older community members or less educated ones [32]. This actually stresses the need for educational campaigns that raise more awareness about HIV and learning to accept PLWHA.

### Practices

Results from this study match those discussed in the literature and continue to revolve around the importance of HIV education. Positive practices were still low among study participants. Thus, this suggests that youth should be provided continuously with HIV education along with the relevant prevention messages, in order to overcome the prevalent misconceptions [33]. In addition to tackling HIV knowledge, policies should be put in place to help reduce negative attitudes. Such policies will help protect high risk populations and secure their rights [34]. Consequently, people in the community will start having a more positive attitude towards PLWHA.

Literature has shown that stigma towards PLWHA affects the quality of life among this population. Moreover, it creates groups of a hidden population that is hard to reach. These groups then find it hard to reach out for treatment and seek care [35]. It is therefore important to have interventions and strategies implemented towards reducing this stigma. One of the major strategies to achieving this is through working on the community level by increasing acceptance towards PLWHA. This is attained by educating people with factual information about the disease [26,36]. Having social support is very important in improving the lives of PLWHA. Positive behavioral change was reported among those who disclosed their HIV status to family and friends, who in return provided them with emotional support [37,38]. Furthermore, changing the attitudes of healthcare workers to non-judgmental, facilitates healthcare seeking behavior among PLWHA and motivates them to communicate and adhere to treatment [37]. An additional way to reduce stigma could also be by having PLWHA talk about their experiences and self-advocate to educate people [38].

Finally, it is noteworthy, that even when participants in this study had high knowledge about HIV transmission and protection, there was still very low awareness regarding HIV situation in Lebanon and the existence of associations working in this field specifically. This again raises and stresses the need of advocacy campaigns targeting youth and helping them know where to reach out to when they need specific information about the topic, or even when they need help if they got infected. Also, it is of major importance for future studies in Lebanon to focus on factors associated with stigma, as all existing ones only focus on the prevalence of HIV among high risk populations.

#### Limitations and strengths

The major limitation of this study was the length and sensitivity of the survey. The questionnaire was too long, which demotivated approached people from participating in the study. Moreover, the sensitivity of the questions played a huge role, where participants felt uncomfortable answering some questions that are related to their sexual behavior, which may have created information bias. This might explain the association we got between illiteracy and positive practices. This is reflected as a social desirability bias, where participants were tending to give answers that are considered more acceptable given our society norms. Also, the random way participants were selected might have created a selection bias and might have thus resulted in some skewness in results and lower representation of different sub-groups. For example, we had a higher number of participants who are highly educated and have high income and lower number of participants who were illiterate. Finally, the relatively low Cronbach alpha value for Practices is another limitation which might have affected the effectiveness of our results. This could mainly be a result of variation across our study objects which might have resulted in such a low value. Therefore, and based on these limitations, we have a couple of suggestions to future studies in similar cultural settings as ours. First, we suggest that the survey be shared online through a link that will further guarantee anonymity of participants. This will decrease the probability of having information bias and will increase participation rate. Second, since respondents always try to align their responses with the purpose of the survey, we advise that researchers introduce the aim of the survey in a friendly way that clearly explains there is no right or wrong answers, thus prompting more honest responses. However, it is important to clarify here that the topic we are trying to tackle is sensitive, and it is impossible to avoid all biases.

However, based on the aforementioned, this study stands out to be unique since it provides an update on the knowledge and attitudes of the general population towards HIV/AIDS and PLWHA in Lebanon. Our study focuses on the general population rather than focusing on the typical studied groups, like medical practitioners and people at risk of acquiring HIV. This gives us an accurate picture of the reality and shows vividly what needs to be tackled in future advocacy campaigns and who to address specifically. For example, and based on our results, it is suggested that future campaigns address people who are from families with lower income and have less access to health services. As such, we suggest that interventions to this group be of dual benefit. Meaning, interventions should offer them educational material and at the same time offer an incentive. This way, the target community will be more enthusiastic to learn about HIV knowing they will be receiving a healthcare service or a food voucher in return. Such interventions should be accompanied by a monitoring and an impact evaluation plan to measure its success. We also suggest that a combination of different interventions be used simultaneously, like having an educational intervention accompanied with a social media campaign intervention. This way we would guarantee a higher impact. Finally, the high Cronbach’s alpha values for total knowledge and attitudes questions, shows that our survey is reliable and is a good tool for measuring the study outcomes.

## Conclusion

Our study emphasizes that there are factors associated with knowledge, attitudes and practices towards PLWHA. Specifically, attitudes towards PLWHA in Lebanon are highly linked to the level of knowledge of its population. Through our results, we have stressed the need for educational programs, advocacy campaigns and policies to help reduce HIV stigma. This will then help start developing interventions and strategies that possibly reduce stigmatization level.

## Supporting information

S1 Table Factor analysis of the HIV knowledge questions. (DOCX)

S2 Table Factor analysis of the HIV transmission questions. (DOCX)

S3 Table Factor analysis of the HIV risk reduction questions. (DOCX)

S4 Table Factor analysis of the HIV attitude questions. (DOCX)

S1 Appendix Survey. (DOCX)

## Abbreviations

HIV: Human Immunodeficiency Virus
AIDS: Acquired Immunodeficiency Syndrome
MENA: Middle East and North Africa
PLWHA: people living with HIV/AIDS
KSA: Kingdom of Saudi Arabia
