# Correction: Knowledge, attitudes and practices towards people living with HIV/AIDS in Lebanon

**DOI:** 10.1371/journal.pone.0259359

**Published:** 2021-10-26

**Authors:** Lara Youssef, Souheil Hallit, Hala Sacre, Pascale Salameh, Michelle Cherfan, Marwan Akel, Mira Hleyhel

There is an error in affiliation 5 for author Pascale Salameh. The correct affiliation 5 is: University of Nicosia Medical School, Nicosia, Cyprus.
