# Supplementary material for: Knowledge, attitudes and practices towards people living with HIV/AIDS in Lebanon
Source: PLoS One. 2021 Mar 25;16(3):e0249025. doi: 10.1371/journal.pone.0249025 (PMC7993853; doi:10.1371/journal.pone.0249025)
Supplement: S1 Table — (DOCX) [file pone.0249025.s001.docx]

Supplementary tables

| Supplementary Table 1. Factor analysis of the HIV knowledge questions. | | | |
| --- | --- | --- | --- |
| Item | Factor 1 | Factor 2 | Factor 3 |
| The virus is the causative agent of HIV/AIDS | 0.481 |  |  |
| HIV and AIDS are the same thing |  | 0.491 |  |
| HIV/AIDS is present in Lebanon |  |  | 0.516 |
| Does AIDS kill? |  |  | 0.851 |
| The current AIDS situation in Lebanon is serious |  | 0.577 |  |
| Is AIDS a sexually transmitted disease? |  |  | 0.724 |
| Can a patient living with HIV/AIDS (PLWHA) still look healthy? |  | 0.484 |  |
| Is HIV/AIDS preventable? |  | 0.375 |  |
| Is a vaccine available on market? |  | 0.593 |  |
| Is HIV/AIDS curable? | 0.759 |  |  |
| Can HIV cause cancer? |  | 0.574 |  |
| Have you ever heard of antiretroviral therapy? |  |  | 0.662 |
| Can people protect themselves from HIV/AIDS by not doing sexual intercourse (or abstinence)? | 0.763 |  |  |
| Can condoms decrease the risk of HIV transmission? |  |  | 0.697 |
| Can HIV infection develop into AIDS within a year? |  | 0.698 |  |
| Does having multiple sexual partners increase the risk of getting HIV infection? |  |  | 0.565 |
| AIDS only affects IV drug users, prostitutes and homosexuals |  |  | 0.795 |
| Is HIV testing mandatory before marriage in Lebanon? | 0.562 |  |  |

KMO=0.71; Bartlett’s p<0.001; Variance explained=47.69%
