# Supplementary material for: Knowledge, attitudes and practices towards people living with HIV/AIDS in Lebanon
Source: PLoS One. 2021 Mar 25;16(3):e0249025. doi: 10.1371/journal.pone.0249025 (PMC7993853; doi:10.1371/journal.pone.0249025)
Supplement: S2 Table — (DOCX) [file pone.0249025.s002.docx]

| Supplementary Table 2. Factor analysis of the HIV transmission questions. | | |
| --- | --- | --- |
| Item | Factor 1 | Factor 2 |
| Unprotected sexual intercourse |  | 0.800 |
| Protected sexual intercourse (using condoms) |  | 0.364 |
| Sexual intercourse while the female is taking oral contraceptives |  | 0.316 |
| Air contact (sneezing or coughing) | 0.616 |  |
| Donating blood |  | 0.725 |
| Mouth kissing | 0.722 |  |
| Hugging |  | 0.631 |
| Sharing bathroom | 0.769 |  |
| Sharing a toilet seat with PLWHA | 0.823 |  |
| Mosquito bites | 0.661 |  |
| Sharing a meal with PLWHA | 0.831 |  |
| Sharing cigarettes with PLWHA | 0.875 |  |
| From a PLWHA pregnant mother to her unborn child |  | 0.745 |
| PLWHA nursing mother to her breastfed baby | 0.440 |  |
| Sharing needles or syringes or sharp objects like razors |  | 0.887 |
| Blood transfusion not screened for HIV |  | 0.840 |
| Shaking hands |  | 0.635 |
| Eating raw meat prepared by PLWHA | 0.727 |  |
| Sharing public swimming pools with PLWHA | 0.818 |  |
| Eating and drinking from the same plate or glass of PLWHA | 0.877 |  |
| Wearing the same clothes of PLWHA | 0.760 |  |
| Promiscuity (sexual habits involving a lot of different partners) |  | 0.743 |
| Dentist tools or instruments |  | 0.642 |

KMO=0.965; Bartlett’s p<0.001; Variance explained=58.82%
