# Supplementary material for: Knowledge, attitudes and practices towards people living with HIV/AIDS in Lebanon
Source: PLoS One. 2021 Mar 25;16(3):e0249025. doi: 10.1371/journal.pone.0249025 (PMC7993853; doi:10.1371/journal.pone.0249025)
Supplement: S3 Table — (DOCX) [file pone.0249025.s003.docx]

| Supplementary Table 3. Factor analysis of the HIV risk reduction questions. | | |
| --- | --- | --- |
| Item | Factor 1 | Factor 2 |
| Having sexual intercourse with only one faithful uninfected partner | 0.662 |  |
| Taking a blood test before marriage | 0.898 |  |
| Using condoms during sexual intercourse | 0.716 |  |
| Abstaining from sexual intercourse | 0.432 |  |
| Relying on Fate and destiny | 0.488 |  |
| Avoiding sex with people who have many sexual partners? | 0.787 |  |
| Avoiding Mosquito bites? |  | 0.687 |
| Avoiding sharing clothes? |  | 0.769 |
| Avoiding breastfeeding baby by PLWHA nursing mother? |  | 0.494 |
| Doing sexual intercourse while the female is taking Oral contraceptives? |  | 0.645 |
| Avoiding using public toilets |  | 0.816 |
| Avoiding sharing food and drink |  | 0.772 |
| Avoiding using polluted/contaminated water |  | 0.825 |
| Screening blood for transfusion | 0.771 |  |
| Use of sterile syringes | 0.832 |  |
| Avoiding sharing sharp objects | 0.800 |  |
| Use of sterile dentist tools and instruments | 0.773 |  |
| Testing for HIV | 0.769 |  |

KMO=0.753; Bartlett’s p<0.001; Variance explained=56.96%
