# Supplementary material for: Knowledge, attitudes and practices towards people living with HIV/AIDS in Lebanon
Source: PLoS One. 2021 Mar 25;16(3):e0249025. doi: 10.1371/journal.pone.0249025 (PMC7993853; doi:10.1371/journal.pone.0249025)
Supplement: S4 Table — (DOCX) [file pone.0249025.s004.docx]

| Supplementary Table 4. Factor analysis of the HIV attitude questions. | | | |
| --- | --- | --- | --- |
| Item | Factor 1 | Factor 2 | Factor 3 |
| I would buy items from a PLWHA shopkeeper or food seller | 0.730 |  |  |
| PLWHA student should be allowed to continue studying at school | 0.814 |  |  |
| PLWHA teacher should be allowed to continue teaching at school | 0.838 |  |  |
| I am willing to share meal with PLWHA | 0.732 |  |  |
| I am willing to work in an office with PLWHA | 0.852 |  |  |
| I am willing to shake hands with PLWHA | 0.801 |  |  |
| I can be a friend with PLWHA | 0.757 |  |  |
| PLWHA children should be educated in separate schools |  |  | 0.377 |
| PLWHA students should be educated in separate universities | 0.453 |  |  |
| PLWHA should be separated from other patients in hospitals and clinics |  |  | 0.596 |
| PLWHA should stay at home, not in hospitals |  | 0.480 |  |
| All healthcare students and professionals should go for mandatory HIV testing |  |  | 0.595 |
| All PLWHA working in healthcare should be dismissed |  |  | 0.492 |
| Physicians should have the right in determining whether to serve PLWHA or not |  | 0.579 |  |
| Physicians should have the right in forcing a medical and/or health procedure (reproductive services) on PLWHA |  |  | 0.560 |
| Provision of ART must be conditional upon the use of contraception in PLWHA |  |  | 0.588 |
| All people should test for HIV before marriage |  |  | 0.561 |
| I feel more fearful in contracting HIV compared to other chronic diseases |  | 0.413 |  |
| Majority of PLWHA are promiscuous |  | 0.764 |  |
| PLWHA should minimize their attendance of public social activities (funfair, concert..) |  | 0.771 |  |
| PLWHA should minimize their attendance of familial activities |  | 0.761 |  |
| PLWHA should minimize their attendance of religious activities |  | 0.675 |  |
| PLWHA should move out of their home and should not live together with their family member |  | 0.648 |  |
| PLWHA should be prohibited from looking after their children who are under 18 years of age |  | 0.729 |  |
| A neighbor who is HIV infected should move away |  |  | 0.518 |
| I feel uncomfortable if I have a neighbor who is PLWHA |  |  | 0.452 |
| PLWHA must be forced to disclose their HIV status to other people |  |  | 0.695 |
| It is necessary to enact a law prohibiting foreign visitors who are PLWHA from visiting Lebanon |  |  | 0.520 |
| A forced disclosure of HIV status on entering another country must be mandatory |  | 0.551 |  |
| It is necessary to enact a law that sexual activity of PLWHA should be criminalized |  | 0.533 |  |
| Insurance companies should refuse PLWHA’s life or health insurance |  |  | 0.395 |
| PLWHA are merely receiving the punishment they deserve |  | 0.677 |  |
| PLWHA should feel ashamed of themselves |  | 0.697 |  |
| PLWHA should feel guilty |  | 0.446 |  |
| PLWHA should feel low self-esteem |  | 0.447 |  |
| PLWHA should feel suicidal |  |  | 0.660 |
| PLWHA should blame themselves |  |  | 0.689 |
| Do you think that PLWHA should blame others | 0.429 |  |  |
| Do you think that PLWHA should be rejected |  | 0.603 |  |
| Do you think that a person who contract HIV through sexual intercourse should be ashamed? |  | 0.424 |  |
| Most PLWHA do not care if they infect other people | 0.311 |  |  |
| Men are to be blamed for the spread of HIV |  | 0.522 |  |

KMO=0.873; Bartlett’s p<0.001; Variance explained=45.55%
