# Supplementary material for: Knowledge, attitudes and practices towards people living with HIV/AIDS in Lebanon
Source: PLoS One. 2021 Mar 25;16(3):e0249025. doi: 10.1371/journal.pone.0249025 (PMC7993853; doi:10.1371/journal.pone.0249025)
Supplement: S1 Appendix — (DOCX) [file pone.0249025.s005.docx]

**Appendix 1.** Survey

1. **SOCIODEMOGRAPHIC**

Age (in years): _______

Gender: 1. Male 2. Female

Marital status: 1.Married 2.Single 3. Divorced 4. Widowed 5. Not reported

Academic year: 0. None 1. Elementary 2.Brevet 3.Baccalaureate 4. Technical Diploma 5.University (no degree) 6. BA/BSc 7.MA/MSc/MBA 8.PhD 9.Not reported

Nationality: 1. Lebanese 2.Other, specify ____________________

Religion: 1. Christian 2. Muslim 3. Druz 4.Other

Working: 0. No 1. Yes

What is your occupation?

Area: 1.Beirut 2. Mount-Lebanon 3.North/Akkar 4. South/Nabatieh 5.Bekaa

Alcohol: 0.No 1. On Occasions 2.< 4x/month 3. 1-3x/wk 4. 4-6x/wk 5. Daily

Smoking: 0. No 1. Occasional Cigarettes 2. Daily Cigarettes 3. Occasional Narguile 4.Frequent Narguile 5. Previous smoker

Family Income: 1.<1000$ 2.Between 1000 & 1500 3.Between 1500 & 3000 4.>3000$

1. **GENERAL KNOWLEDGE:**

| **Level of knowledge of HIV/AIDS** | **1.Yes** | **0.No** | **2. Don’t know** |
| --- | --- | --- | --- |
| 1. The virus is the causative agent of HIV/AIDS |  |  |  |
| 1. HIV and AIDS are the same thing |  |  |  |
| 1. HIV/AIDS is present in Lebanon |  |  |  |
| 1. Does AIDS kill? |  |  |  |
| 1. The current AIDS situation in Lebanon is serious |  |  |  |
| 1. Is AIDS a sexually transmitted disease? |  |  |  |
| 1. Can a **patient living with HIV/AIDS (PLWHA)** still look healthy? |  |  |  |
| 1. Is HIV/AIDS preventable? |  |  |  |
| 1. Is a vaccine available on market? |  |  |  |
| 1. Is HIV/AIDS curable? |  |  |  |
| 1. Can HIV cause cancer? |  |  |  |
| 1. Have you ever heard of antiretroviral therapy? |  |  |  |
| 1. Can people protect themselves from HIV/AIDS by not doing sexual intercourse (or abstinence)? |  |  |  |
| 1. Can condoms decrease the risk of HIV transmission? |  |  |  |
| 1. Can HIV infection develop into AIDS within a year? |  |  |  |
| 1. Does having multiple sexual partners increase the risk of getting HIV infection? |  |  |  |
| 1. AIDS only affects IV drug users, prostitutes and homosexuals |  |  |  |
| 1. Is HIV testing mandatory before marriage in Lebanon? |  |  |  |
| 1. Do you believe that HIV/AIDS is a serious disease? |  |  |  |
| 1. Despite receiving a negative HIV test result, a person could still be infected with HIV |  |  |  |

1. **MODE OF TRANSMISSION:**

| **HIV can be passed from one person to another by:** | **1.Yes** | **0. No** | **2.Don’t know** |
| --- | --- | --- | --- |
| 1. Unprotected sexual intercourse |  |  |  |
| 2.Protected sexual intercourse (using condoms) |  |  |  |
| 3.Sexual intercourse while the female is taking oral contraceptives |  |  |  |
| 4.Air contact (sneezing or coughing) |  |  |  |
| 5.Donating blood |  |  |  |
| 6.Mouth kissing |  |  |  |
| 7.Hugging |  |  |  |
| 8.Sharing bathroom |  |  |  |
| 9.Sharing a toilet seat with PLWHA |  |  |  |
| 10.Mosquito bites |  |  |  |
| 11.Sharing a meal with PLWHA |  |  |  |
| 12.Sharing cigarettes with PLWHA |  |  |  |
| 13.From a PLWHA pregnant mother to her unborn child |  |  |  |
| 14.PLWHA nursing mother to her breastfed baby |  |  |  |
| 15.Sharing needles or syringes or sharp objects like razors |  |  |  |
| 16.Blood transfusion not screened for HIV |  |  |  |
| 17.Shaking hands |  |  |  |
| 18.Eating raw meat prepared by PLWHA |  |  |  |
| 19.Sharing public swimming pools with PLWHA |  |  |  |
| 20.Eating and drinking from the same plate or glass of PLWHA |  |  |  |
| 21.Wearing the same clothes of PLWHA |  |  |  |
| 22.Promiscuity (sexual habits involving a lot of different partners) |  |  |  |
| 23.Dentist tools or instruments |  |  |  |

| **HIV transmission can be avoided or have its risk reduced by:** | **1.Yes** | **0.No** | **2.Don’t know** |
| --- | --- | --- | --- |
| 1. Having sexual intercourse with only one faithful uninfected partner |  |  |  |
| 2. Taking a blood test before marriage |  |  |  |
| 3.Using condoms during sexual intercourse |  |  |  |
| 4.Abstaining from sexual intercourse |  |  |  |
| 5. Relying on Fate and destiny |  |  |  |
| 6.Avoiding sex with people who have many sexual partners? |  |  |  |
| 7.Avoiding Mosquito bites? |  |  |  |
| 8.Avoiding sharing clothes? |  |  |  |
| 9.Avoiding breastfeeding baby by PLWHA nursing mother? |  |  |  |
| 10.Doing sexual intercourse while the female is taking Oral contraceptives? |  |  |  |
| 11.Avoiding using public toilets |  |  |  |
| 12.Avoiding sharing food and drink |  |  |  |
| 13.Avoiding using polluted/contaminated water |  |  |  |
| 14.Screening blood for transfusion |  |  |  |
| 15.Use of sterile syringes |  |  |  |
| 16.Avoiding sharing sharp objects |  |  |  |
| 17.Use of sterile dentist tools and instruments |  |  |  |
| 18.Testing for HIV |  |  |  |
| 19.Being vaccinated for HIV |  |  |  |

1. **ATTITUDE/DISCRIMINATION TOWARDS PLWHA**

| **Attitude/Discrimination towards PLWHA** | **1.Strongly disagree** | **2.Disagree** | **3.Agree** | **4.Strongly agree** |
| --- | --- | --- | --- | --- |
| 1.I would buy items from a PLWHA shopkeeper or food seller |  |  |  |  |
| 2. PLWHA student should be allowed to continue studying at school |  |  |  |  |
| 3. PLWHA teacher should be allowed to continue teaching at school. |  |  |  |  |
| 4.I am willing to share meal with PLWHA |  |  |  |  |
| 5.I am willing to work in an office with PLWHA |  |  |  |  |
| 6.I am willing to shake hands with PLWHA |  |  |  |  |
| 7.I can be a friend with PLWHA |  |  |  |  |
| 8.PLWHA children should be educated in separate schools |  |  |  |  |
| 9.PLWHA students should be educated in separate universities |  |  |  |  |
| 10.PLWHA should be separated from other patients in hospitals and clinics |  |  |  |  |
| 11.PLWHA should stay at home, not in hospitals |  |  |  |  |
| 12.All healthcare students and professionals should go for mandatory HIV testing |  |  |  |  |
| 13.All PLWHA working in healthcare should be dismissed |  |  |  |  |
| 14.Physicians should have the right in determining whether to serve PLWHA or not |  |  |  |  |
| 15.Physicians should have the right in forcing a medical and/or health procedure (reproductive services) on PLWHA |  |  |  |  |
| 16.Provision of ART must be conditional upon the use of contraception in PLWHA |  |  |  |  |
| 17.All people should test for HIV before marriage |  |  |  |  |
| 18.I feel more fearful in contracting HIV compared to other chronic diseases |  |  |  |  |
| 19.Majority of PLWHA are promiscuous |  |  |  |  |
| 20.PLWHA should minimize their attendance of public social activities (funfair, concert..) |  |  |  |  |
| 21.PLWHA should minimize their attendance of familial activities |  |  |  |  |
| 22.PLWHA should minimize their attendance of religious activities |  |  |  |  |
| 23.PLWHA should move out of their home and should not live together with their family member |  |  |  |  |
| 24.PLWHA should be prohibited from looking after their children who are under 18 years of age |  |  |  |  |
| 25.A neighbor who is HIV infected should move away |  |  |  |  |
| 26.I feel uncomfortable if I have a neighbor who is PLWHA |  |  |  |  |
| 27.PLWHA must be forced to disclose their HIV status to other people |  |  |  |  |
| 28.It is necessary to enact a law prohibiting foreign visitors who are PLWHA from visiting Lebanon |  |  |  |  |
| 29.A forced disclosure of HIV status on entering another country must be mandatory |  |  |  |  |
| 30.It is necessary to enact a law that sexual activity of PLWHA should be criminalized |  |  |  |  |
| 31.Insurance companies should refuse PLWHA’s life or health insurance |  |  |  |  |
| 32.PLWHA are merely receiving the punishment they deserve |  |  |  |  |
| 33.PLWHA should feel ashamed of themselves |  |  |  |  |
| 34.PLWHA should feel guilty |  |  |  |  |
| 35.PLWHA should feel low self-esteem |  |  |  |  |
| 36.PLWHA should feel suicidal |  |  |  |  |
| 37.PLWHA should blame themselves |  |  |  |  |
| 38.Do you think that PLWHA should blame others |  |  |  |  |
| 39.Do you think that PLWHA should be rejected |  |  |  |  |
| 40.Do you think that a person who contract HIV through sexual intercourse should be ashamed? |  |  |  |  |
| 41.Most PLWHA do not care if they infect other people |  |  |  |  |
| 42.Men are to be blamed for the spread of HIV |  |  |  |  |
| 43.Quarantine is the best way to prevent HIV |  |  |  |  |
| 44.PLWHA must choose not to marry |  |  |  |  |
| 45.PLWHA must choose not to have sexual intercourse |  |  |  |  |
| 46.PLWHA must decide not to have (more) children |  |  |  |  |
| 47.PLWHA would not make other colleagues apprehensive |  |  |  |  |
| 48.If you become infected with HIV, your life will become over |  |  |  |  |
| 49. PLWHA women have the right to become pregnant |  |  |  |  |
| 50.Do you think that it can be appropriate to sterilize a woman PLWHA, even if this is not her choice |  |  |  |  |
| 51.PLWHA must be the subject of gossip and target of verbal and physical insult, harassment and threats |  |  |  |  |
| 52.PLWHA must not have human rights and fundamental freedom declared in the governmental laws and policies |  |  |  |  |

1. **PRACTICES RELATED TO HIV/AIDS**

**The answers are completely anonymous and confidential.**

|  | **1.Yes** | **0.No** | **2.Don’t know** | **3. Not applicable** |
| --- | --- | --- | --- | --- |
| 1.I have had sexual intercourse before |  |  |  |  |
| 2.I have done the HIV test before |  |  |  |  |
| 3.If yes, how many times have you been tested? |  |  |  |  |
| 4.If yes, why have you been tested? |  |  |  |  |
| 5.I have done the HIV test in the past 12 months |  |  |  |  |
| 6.I know my HIV status |  |  |  |  |
| 7.If you were HIV positive individual, whom will you tell? |  |  |  |  |
| 8.I have used injectable illegal drugs |  |  |  |  |
| 9.I share needles with other drug users |  |  |  |  |
| 10.The last three times I had sexual intercourse, it was with the same partner |  |  |  |  |
| 11.I usually use a condom during sexual intercourse |  |  |  |  |
| 12.I use a condom regularly during sexual intercourse with occasional partner |  |  |  |  |
| 13.I have used a condom during the last sexual intercourse |  |  |  |  |
| 14.I have had sex under the influence of alcohol |  |  |  |  |

1. **HIV/AIDS AWARENESS IN LEBANON**

| **Awareness about HIV/AIDS in Lebanon** | **1.Yes** | **0.No** |
| --- | --- | --- |
| 1. Have you ever discussed HIV/AIDS related topics with your parents? |  |  |
| 1. Have you ever discussed HIV/AIDS related topics with your friends? |  |  |
| 1. Do you know anyone with HIV/AIDS? |  |  |
| 1. Do you know someone who has died from HIV/AIDS? |  |  |
| 1. Do you discuss the risks with your partner? |  |  |
| 1. Do you think HIV/AIDS topic is still a taboo in Lebanon? |  |  |
| 1. Would you seek counseling and advice if you suspected having HIV/AIDS |  |  |
| 1. My professional education has provided me with enough education/ information to work safely with PLWHA. |  |  |
| 1. Using condoms is necessary during sexual intercourse with occasional partner |  |  |
| 1. Everyone should ask their partner to test for HIV before the first sexual intercourse |  |  |
| 1. Do you believe that HIV/AIDS campaigns are adequately/frequently made in Lebanon? |  |  |
| 1. Do you think more awareness campaigns on HIV/AIDS are needed among the Lebanese population? |  |  |
| 1. Do you think we should advocate for rights of PLWHA |  |  |
| 1. Do you think that we should offer emotional, physical and referral support for PLWHA |  |  |
| 1. Have you ever checked the website of the Ministry of Public Health of Lebanon for HIV/AIDS information? |  |  |
| 1. Do you know any associations for HIV/AIDS in Lebanon? |  |  |
| 1. If you know associations for HIV/AIDS, please mention them: |  | |
